# A Multi-Component Prime-Boost Vaccination Regimen with a Consensus MOMP Antigen Enhances *Chlamydia trachomatis* Clearance

**DOI:** 10.3389/fimmu.2016.00162

**Published:** 2016-04-28

**Authors:** Alexander Badamchi-Zadeh, Paul F. McKay, Bette T. Korber, Guillermo Barinaga, Adam A. Walters, Alexandra Nunes, João Paulo Gomes, Frank Follmann, John S. Tregoning, Robin J. Shattock

**Affiliations:** ^1^Mucosal Infection and Immunity Group, Imperial College London, London, UK; ^2^Los Alamos National Laboratory, Theoretical Division, Los Alamos, NM, USA; ^3^Department of Infectious Diseases, National Institute of Health, Lisbon, Portugal; ^4^Chlamydia Vaccine Research, Department of Infectious Disease Immunology, Statens Serum Institute, Copenhagen, Denmark

**Keywords:** *Chlamydia trachomatis*, consensus, mosaic, prime-boost regimens, adenovirus-vector vaccines, MVA-vector vaccines, DNA vaccines

## Abstract

**Background:**

A vaccine for *Chlamydia trachomatis* is of urgent medical need. We explored bioinformatic approaches to generate an immunogen against *C. trachomatis* that would induce cross-serovar T-cell responses as (i) CD4^+^ T cells have been shown in animal models and human studies to be important in chlamydial protection and (ii) antibody responses may be restrictive and serovar specific.

**Methods:**

A consensus antigen based on over 1,500 major outer membrane protein (MOMP) sequences provided high epitope coverage against the most prevalent *C. trachomatis* strains *in silico*. Having designed the T-cell immunogen, we assessed it for immunogenicity in prime-boost regimens. This consensus MOMP transgene was delivered using plasmid DNA, Human Adenovirus 5 (HuAd5) or modified vaccinia Ankara (MVA) vectors with or without MF59^®^ adjuvanted recombinant MOMP protein.

**Results:**

Different regimens induced distinct immune profiles. The DNA-HuAd5-MVA-Protein vaccine regimen induced a cellular response with a Th1-biased serum antibody response, alongside high serum and vaginal MOMP-specific antibodies. This regimen significantly enhanced clearance against intravaginal *C. trachomatis* serovar D infection in both BALB/c and B6C3F1 mouse strains. This enhanced clearance was shown to be CD4^+^ T-cell dependent. Future studies will need to confirm the specificity and precise mechanisms of protection.

**Conclusion:**

A *C. trachomatis* vaccine needs to induce a robust cellular response with broad cross-serovar coverage and a heterologous prime-boost regimen may be an approach to achieve this.

## Introduction

Genital chlamydial infection is the most common cause of bacterial sexually transmitted diseases (STDs) worldwide, accounting for more than 90 million cases of STDs globally each year (1). Over US$3 billion is spent annually on an estimated four million reported clinical cases of genital chlamydial infections in the US alone (2), thus development of a vaccine against *Chlamydia trachomatis* represents a significant public health priority. A promising vaccine antigen of *C. trachomatis* is the major outer membrane protein (MOMP). The MOMP antigen has been used in a range of previous pre-clinical vaccine studies with a mixture of encouraging (3, 4) and disappointing results (5, 6). This may reflect the high level of amino acid variability in the MOMP antigen, the basis for *C. trachomatis* serotypes (7).

New bioinformatic approaches have been developed to account for amino acid diversity and aid in the design of immunogens to induce cross-serovar T-cell responses: these include the design of mosaic or conserved antigen sequences. Mosaic vaccine antigens are designed with the intent to be used in polyvalent combinations to optimize the coverage of potential T-cell epitopes (8), for example, HIV mosaic antigens increased the breadth and potency of vaccine-elicited immune responses (9), conferring protective responses in non-human primate models (10). Consensus vaccine antigens rely on a single centralized antigen designed to reduce sequence distances between the vaccine and circulating strains by using the most common amino acid at each position of the protein (11). By reducing the genetic differences between the vaccine and the primary isolate, consensus antigens can increase the breadth of immune response (12).

Here, we computationally design and *in silico* assess both consensus and mosaic MOMP antigens for broad T-cell coverage against the *C. trachomatis* genital serovars D–K, for the reasons that (a) chlamydial infections in both animal models and humans suggest a strong protective role for CD4^+^ Th1-biased immune responses (4, 13, 14) and (b) that these may be supplemented by MOMP-specific antibodies able to mediate antibody-dependent cellular cytotoxicity (ADCC) (15).

We assess the quantity and quality of the antibody and cellular response to MOMP following different prime-boost combinations using DNA vaccines, recombinant viral vectors, and protein-in-adjuvant formulations (16). Both DNA and recombinant viral vectors preferentially induce cellular immunity (17) and subunit proteins humoral immunity: the use of different prime-boost combinations can be tailored to influence the phenotype of both arms of induced adaptive immunity. DNA vaccine vectors, human Adenovirus 5 (HuAd5), and modified vaccinia Ankara (MVA) viral vectors have already entered phase I clinical trials, showing safety and tolerability (18, 19). Research groups have reported enhanced T-cell induction through heterologous prime-boost vaccination strategies in a range of disease models [including tuberculosis (20), HIV (21), HPV (22), and Ebola (23)] but these strategies have yet to be comprehensibly investigated for *C. trachomatis*.

We demonstrate that different vaccination regimens when used to deliver the same MOMP antigen via differing platforms can be configured to induce distinct immune outcomes. We further investigate which distinct MOMP-specific immune responses are required for enhanced *C. trachomatis* clearance following genital challenge in mice. We observe that a regimen using DNA-HuAd5-MVA-Protein (DAMP) vaccines reduced bacterial load early after infection regardless of mouse strain used, and that this enhanced clearance while dependent upon CD4^+^ T-cell responses may have been augmented by induced MOMP-specific antibody responses.

## Materials and Methods

### Bioinformatic Antigen Design

One thousand four hundred sixty-four serovar E *ompA* sequences, surveyed from a total of 5,026 *C. trachomatis* strains isolated in 33 distinct geographic regions from five continents were compared (24). Phylogenetic analysis was based on the protein sequence alignment derived from Ref. (24), and the maximum likelihood tree was created using FastTree (25) using default settings; the figure was generated using Rainbow Tree (www.hiv.lanl.gov). Forty-nine distinct *ompA* variants were present within the serovar E sequences, and were used in the *in silico* generation of the consensus MOMP (Con E) antigen sequence for the experimental studies. The generated consensus and mosaic antigens were assessed for their coverage against different *Chlamydia* MOMP variants using the Epitope Coverage Assessment Tool EPICOVER. Full bioinformatic tool settings, a detailed description of the method, and consensus and mosaic antigen sequence information is provided in the File S1 in Supplementary Material.

### Plasmid, Viral Vectors, and Recombinant Protein

Mammalian codon optimized MOMP Con E antigen was synthesized by GeneArt (Invitrogen, UK) and cloned into pcDNA3.1 (Invitrogen, UK). Con E was homologously recombined into the E1 and E3 deleted HuAd5 genome plasmid, pAL1112 (kindly provided by Prof. Gavin Wilkinson, Cardiff University). Con E was recombined into the MVA pox vector by the Viral Vector Core Facility, The Jenner Institute (University of Oxford, UK). *Escherichia coli* codon optimized Con E expressed at too low a yield in BL21 *E. coli*, and as such a recombinant MOMP matching *C. trachomatis* from serovar D/UW/Cx expressed in BL21 *E. coli* was used.

### *Chlamydia* 

*Chlamydia trachomatis* serovar D (strain UW-3/Cx) was provided by Dr. Frank Follmann (Statens Serum Institut) and propagated in McCoy cells as described previously (26). Chlamydial EBs were harvested, purified, and quantified as described in Ref. (26), and stored at −80°C in SPG buffer (Sucrose/Phosphate/Glutamic acid: 0.2 M sucrose, 20 mM sodium phosphate, and 5 mM glutamic acid).

### Mice Immunizations and Infections

Female 6–8 weeks old BALB/c mice (Harlan, Stornoway, UK) and female 6–8 weeks old B6C3F1 mice (Charles River, Italy) were kept in specific-pathogen-free conditions in accordance with the UK Home Office guidelines. All work was approved by the Imperial College Ethical Review Process (ERP) Committee. Mice received immunizations at 3-week intervals (Table 1). DNA vaccinations were at 10 μg doses, intramuscularly into the hind quadriceps muscle in a volume of 50 μl with electroporation. Electroporation was with 5 mm electrodes at the immunization site using an ECM 830 Square Wave Electroporation System (BTX), with three pulses of 100 V each, followed by three pulses of the opposite polarity with each pulse (P_ON_) lasting 50 ms and an interpulse (P_OFF_) interval of 50 ms. All HuAd5 and MVA vaccinations were at dosages of 10^7^ PFU and 10^6^ PFU, respectively. rMOMP was administered at a dose of 10 μg in a 1:1 mixture with MF59^®^ (an oil-in-water emulsion adjuvant) (Novartis, Sienna, Italy) in a final volume of 50 μl for intramuscular immunizations. Due the multiple components within the immunization regimens, vehicle and vector-alone controls were not included to reflect reduce, replace and refinement practice. Seven days prior to intravaginal infection, mice were injected subcutaneously with 2 mg of DMPA (Depo-Provera, Pfizer). For intravaginal infections, purified *C. trachomatis* D/UW-3/Cx EBs were dissolved in SPG buffer to a concentration of 4 × 10^7^ IFU/ml, mice were anesthetized, and 10 μl of the EB solution pipetted into the mouse vagina. The optimal infective dose of 4 × 10^5^ IFU of *C. trachomatis* D/UW-3/Cx EBs per mouse was previously determined by Dr. Frank Follmann, SSI (unpublished data) and consistent with Ref. (27, 28). Furthermore, bacterial clearance profiles were consistent between naive BALB/c and B6C3F1 mice at this infective dose (Figure S2 in Supplementary Material). Unfortunately *C. trachomatis* E/Bour could not be propagated to a high enough infectious titer for intravaginal infection.

**Table 1:** **Multi-component prime-boost vaccine regimens**.

Regimen	d0	d21	d42	d63	d84
DDDAM	DNA	DNA	DNA	HuAd5	MVA
DDDA	DNA	DNA	DNA	HuAd5	
DDDM	DNA	DNA	DNA	MVA	
DDD	DNA	DNA	DNA		
AM	HuAd5	MVA			
DAMP	DNA	HuAd5	MVA	Protein	
AMPP	HuAd5	MVA	Protein	Protein	
DDPP	DNA	DNA	Protein	Protein	
APP	HuAd5	Protein	Protein		
PPP	Protein	Protein	Protein		
PP	Protein	Protein			

*In vaccine regimen nomenclature, D represents DNA (+ electroporation), A represents HuAd5, M represents MVA, and P represents recombinant MOMP protein adjuvanted with MF59^®^*.

### Intravaginal *C. trachomatis* Load Quantification

Vaginal swabs were obtained at 3, 7, 10, and 14 days after infection. Swabs were vortexed with glass-beads in 500 μl SPG buffer and stored at −80°C until analysis. Infectious load was determined as described in Ref. (29). Inclusions were visualized by staining with polyclonal rabbit anti-MOMP serum (provided by Dr. Frank Follmann, SSI), followed by an Alexa 594-conjugated goat anti-rabbit H + L (Life Technologies, UK).

### Mice Sampling

Tail bleeds were collected before regimen, and 2 weeks post each immunization. Blood was collected and centrifuged at 1,000 × *g* for 10 min. The serum was harvested and stored at −20°C. To assess IFN-γ T-cell responses, lymphocyte cultures from spleens were prepared as described previously (16). Vaginal lavage was performed at the same time points as tail bleeds, using three 25 μl washes/mouse with sterile phosphate buffered saline (PBS) that were later pooled. Lavage samples were incubated with protease inhibitor (Roche Diagnostics, Germany) before centrifuging at 1,000× *g* for 10 min. The fluid supernatant from these samples was harvested and stored at −20°C.

### Semi-Quantitative MOMP-Specific ELISA, Avidity Assay, and MOMP-Specific IFN-γ ELISpot

A semi-quantitative immunoglobulin ELISA protocol described previously (30) was followed. The avidity indices of serum samples were determined by their antibody–antigen binding resistance to 8 M urea. Serum samples were pre-diluted to give an OD_450 nm_ readout between 1.0 and 1.5 in an ELISA and were added to MOMP antigen coated plates. Plates were then washed three times with either PBS-T or 8 M urea in PBS-T, before incubating with anti-mouse IgG-HRP. Samples were developed with TMB as described above. The avidity index was calculated as the percentage of urea treated OD_450 nm_/PBS-T OD_450 nm_. IFN-γ ELISpot assays (Mabtech, UK) were carried out on mouse splenocytes as to manufacturer’s instructions.

### Depletion of CD4^+^ T Cells

Mice were depleted of CD4^+^ T cells by the i.p. route with injections of 500 μg monoclonal anti-mouse CD4 IgG2b (clone GK1.5) (BioXcell, Cat: BE0003-1) on days −1 and +1 with respect to day of challenge being day 0. The depletion of CD4^+^ T cells was verified by FACS analysis on murine PBMC, splenocytes, and vaginal tissue on day +2 using anti-CD3e PE, anti-CD4 APC, and anti-CD8a eFluor605NC antibodies (All BD Biosciences, UK).

### Statistical Analysis

All statistical analyses were carried out using Prism 6.0 (GraphPad, CA, USA). Normality of the data distribution was assessed using the Kolmogorov–Smirnov normality test. For non-parametric data, the Kruskal–Wallis test with Dunn’s multiple comparison post-test was used to compare more than two groups, or the two-tailed Mann–Whitney test to compare two groups. For parametric data, a one-way ANOVA was used for multiple comparisons, with Bonferroni’s multiple comparison post-test for comparison of specific groups. *P* < 0.05 was considered significant (*p < 0.05, **p < 0.01, and ***p < 0.001).

## Results

### Design and Cross-Serovar Coverage Assessment of Consensus and Mosaic MOMP Antigens

**Figure 1:**
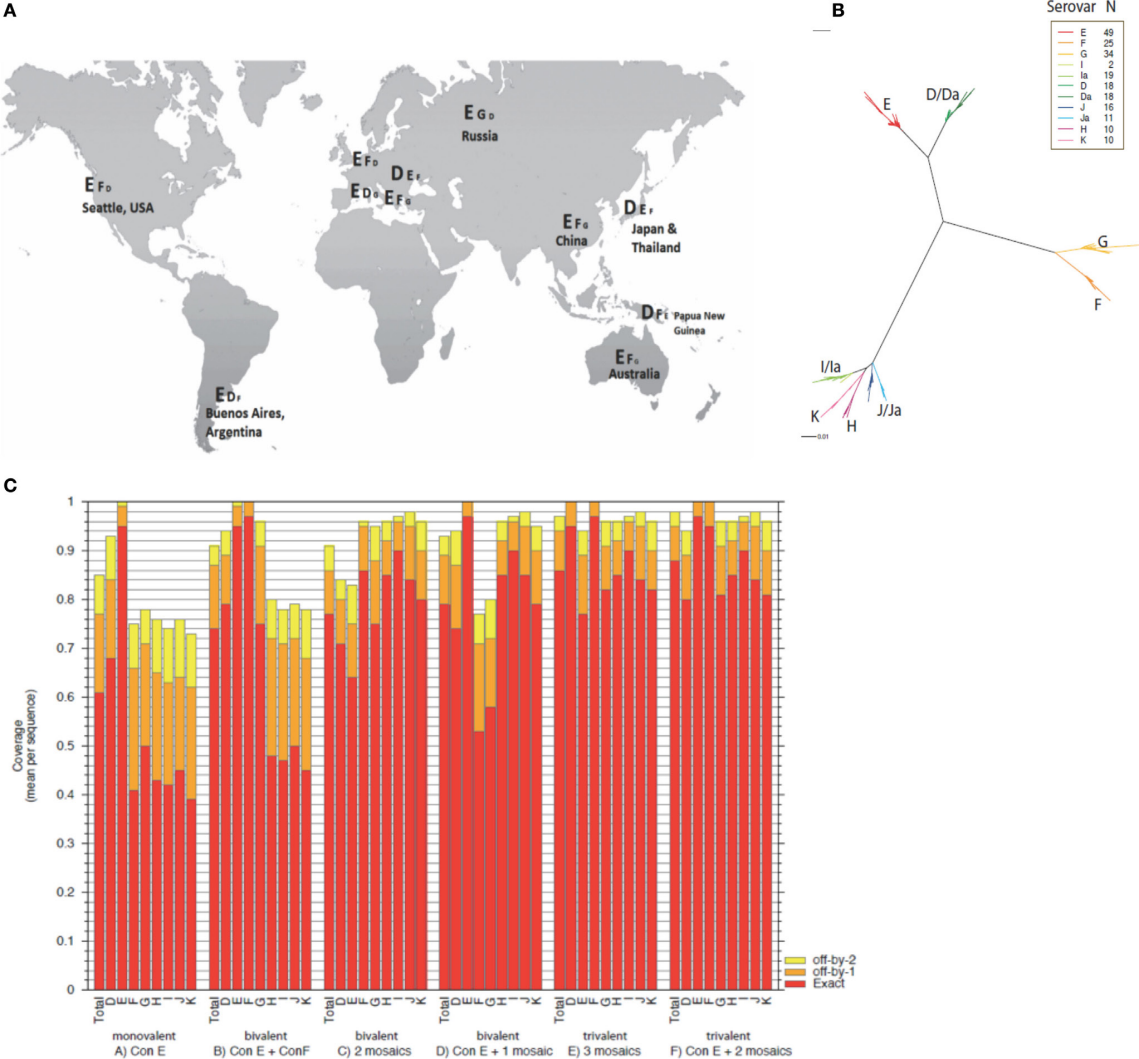
**Global *C. trachomatis* serovar prevalence, phylogeny, and theoretical epitope coverage of consensus and mosaic MOMP antigens**. **(A)** The serovar prevalence of *C. trachomatis* worldwide compiled from a literature review and represented in descending prevalence at global locations. **(B)** A phylogenetic maximum likelihood tree based on the *ompA* sequence alignments derived from Ref. (24) was created using FastTree and the graphic generated by Rainbow Tree. **(C)** Potential epitope coverage against all serovars (total) and individual serovars (serovars D–K) were analyzed for a monovalent Con E antigen, Con E and Con F antigens, two mosaic antigens, a Con E antigen and a mosaic antigen, three mosaic antigens, and a Con E antigen with two additional mosaic antigens using EPICOVER. Mean 9-mer coverage presented against individual and total combined serovars D–K, with exact (red), off-by-1 (orange), and off-by-2 (yellow) epitope matching.

The worldwide prevalence of specific urogenital *C. trachomatis* serovars has not been fully characterized. A literature review (PubMed) identified 13 publications describing the country or regional serovar-specific prevalence (31–43). In 10 out of the 13 *C. trachomatis* serovar surveys published, serovar E emerged as the most prevalent (Figure 1A). MOMP is a lead vaccine antigen candidate for *C. trachomatis*, and with worldwide MOMP sequence data available (24) for the differing genital serovars (D–K, Da, Ia, and Ja) it was possible to bioinformatically perform MOMP-based immunogen design. We found sequences within all serovars to be conserved, with only sporadic amino acid substitutions (Figure 1B). From our phylogenetic analyses, *C. trachomatis* has quite distant species, but high levels of conservation within a serovar. Thus, this kind of phylogenetic profile lends itself more toward a consensus antigen design approach as opposed to a single mosaic antigen design (8). Based on 49 published serovar E sequence variants (24), we generated a novel MOMP consensus sequence (Con E), which fully matched the solution for a single mosaic and was identical to the circulating *C. trachomatis* strain E-Bour, as well as to eight additional partial MOMP protein sequences from a wide variant of geographic origins (24). Using a single consensus antigen, the potential epitope coverage of the E serovar led to extremely high coverage (~95%) (Figure 1C). For even broader coverage, our analyses suggest that multiple mosaic antigens may be more appropriate (Figure 1C; File S1 in Supplementary Material).

### Differences in Humoral and Cellular Immunogenicity of *C. trachomatis* MOMP Vaccines Following Intramuscular Multi-Component Prime-Boost Regimen Screen

**Figure 2:**
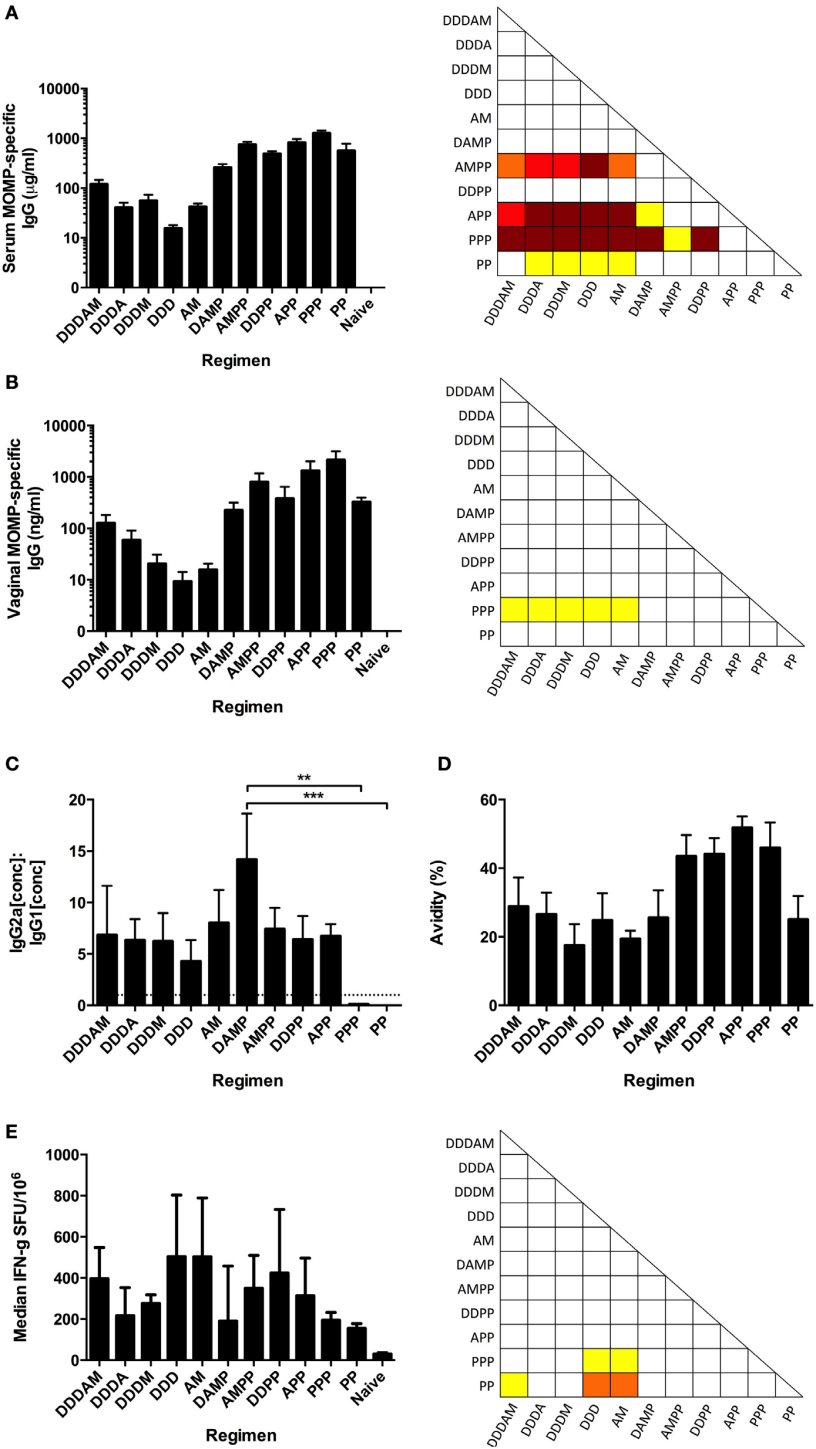
**Antibody and cellular responses following multi-component prime-boost vaccination regimens**. BALB/c mice (*n* = 8 per group) were intramuscularly immunized in various prime-boost regimens, with sera and vaginal wash collected 2 weeks after final boost. MOMP-specific IgG concentrations were measured in serum **(A)** and vaginal washes **(B)**, expressed as the mean + SEM concentrations. **(C)** Serum MOMP-specific IgG2a and IgG1 isotype concentrations were measured by ELISA, and the mean + SEM IgG2a:IgG1 ratios plotted. The dotted line indicates the IgG2a:IgG1 ratio of 1, demonstrating Th1-skewing above this line or Th2-skewing below it. **(D)** Serum antibody avidity was measured by MOMP-specific IgG ELISA with non-reducing (H_2_O) and reducing (8 M urea) washes after sample addition. Results are shown as percentage (%) change in binding (reducing OD_450_/non-reducing OD_450_ × 100). IgG concentrations, avidities, and IgG2a:IgG1 ratio represented as group means and SEM. **(E)** Vaccinated BALB/c mice (*n* = 8 per group) were sacrificed 1 week post-final immunization and splenocytes assessed by IFN-γ ELISpot for MOMP-reactive T cells stimulated by a peptide pool consisting of 15-mers overlapping by 11 amino acids. Data expressed as group medians (+ interquartile range) (SFU/million antigen stimulated cells). *p ≤ 0.05 (yellow), **p ≤ 0.005 (orange), ***p ≤ 0.0005 (red), and ****p ≤ 0.0001 (dark red) by one-way ANOVA with Bonferroni’s multiple comparison post-test on logged values **(A–D)** and by Kruskal–Wallis with Dunn’s multiple comparison test **(E)**.

The immunogenicity of the consensus MOMP antigen was assessed in BALB/c mice immunized in multi-component prime-boost regimens with DNA (D), HuAd5 (A), MVA (M), and protein with the oil-in-water emulsion adjuvant MF59^®^ (P) vaccines (Table 1). The MF59^®^ adjuvant has been demonstrated to induce IL-5 and IL-10 responses to the MOMP antigen and was, therefore, used as a comparator to the more Th1-skewing DNA and viral vector approaches (44). Serum and vaginal washes were sampled 2 weeks after final immunization. The highest MOMP-specific serum IgG concentrations were observed after the PPP regimen (mean + SEM = 1.27 ± 0.16 mg/ml) and the lowest from the DDD regimen (mean + SEM = 15.4 ± 2.54 μg/ml) (Figure 2A). Protein (+ MF59^®^) immunization significantly increased MOMP-specific serum IgG concentrations compared to prime-boost regimen without two protein boosts. MOMP-specific vaginal IgG concentrations were measured following the multi-component prime-boost regimens (Figure 2B). The PPP regimen induced significantly higher MOMP-specific vaginal IgG than DDDAM, DDDA, DDDM, DDD, AM, or the naive group (*p* ≤ 0.05). MOMP-specific IgA was not detectable in the sera or vaginal washes following any of the prime-boost regimens (data not shown).

Viral vector vaccines have been shown to induce high and long-lasting cytophilic, Th1 skewed, antibody responses (45). A correlate for Th1 and Th2 skewing of the immune response in mice is the antigen-specific IgG2a to IgG1 ratio. MOMP-specific serum IgG2a and IgG1 concentrations were measured by ELISA, and their ratios calculated (Figure 2C). The highest MOMP-specific serum IgG2a:IgG1 ratio was induced following the DAMP regimen (mean = 14.2), with the lowest ratio induced following PP (+ MF59^®^) vaccination (mean = 0.0036). There was a significant statistical difference in the IgG2a:IgG1 ratios between DAMP and PPP (*p* ≤ 0.005) and DAMP and PP (*p* ≤ 0.0005, one-way ANOVA with Bonferroni’s multiple comparison post-test). Protein-only vaccine regimens induced Th2-biased immune environments indicated by IgG2a:IgG1 ratios of <1 caused by high IgG1 concentrations. Vaccine regimens with a vector-based vaccine prime consistently induced a Th1-biased, cytophilic antibody response as indicated by IgG2a:IgG1 ratios of >1.

To differentiate and qualitatively evaluate the humoral responses, an avidity assay was performed (Figure 2D). All protein-free regimens had mean avidity indices of <30%, with the DAMP and PP regimens also having low mean avidity indices. Regimens involving two protein boosts all had avidity indices >40%, with the APP regimen inducing MOMP-specific serum IgGs with the highest avidity (mean = 51.8%).

T-cell responses were assessed 1 week after the final immunization. MOMP-specific IFN-γ + T-cell responses as assessed by ELISpot were induced by all prime-boost regimens, with the DDD and AM regimens inducing the strongest T-cell responses (a median of 504 and 502 SFU/10^6^ splenocytes, respectively, Figure 2E). T-cell responses induced by the DDD and AM regimens were significantly higher than those induced in the PP regimen (*p* ≤ 0.05). From this, we conclude that there were significant differences in both the quantity and quality of the antibody and cellular response following the different regimens.

### Multi-Component Prime-Boost Regimens Induce Analogous Immunogenicity Profiles in Both BALB/c and B6C3F1 Mice

**Figure 3:**
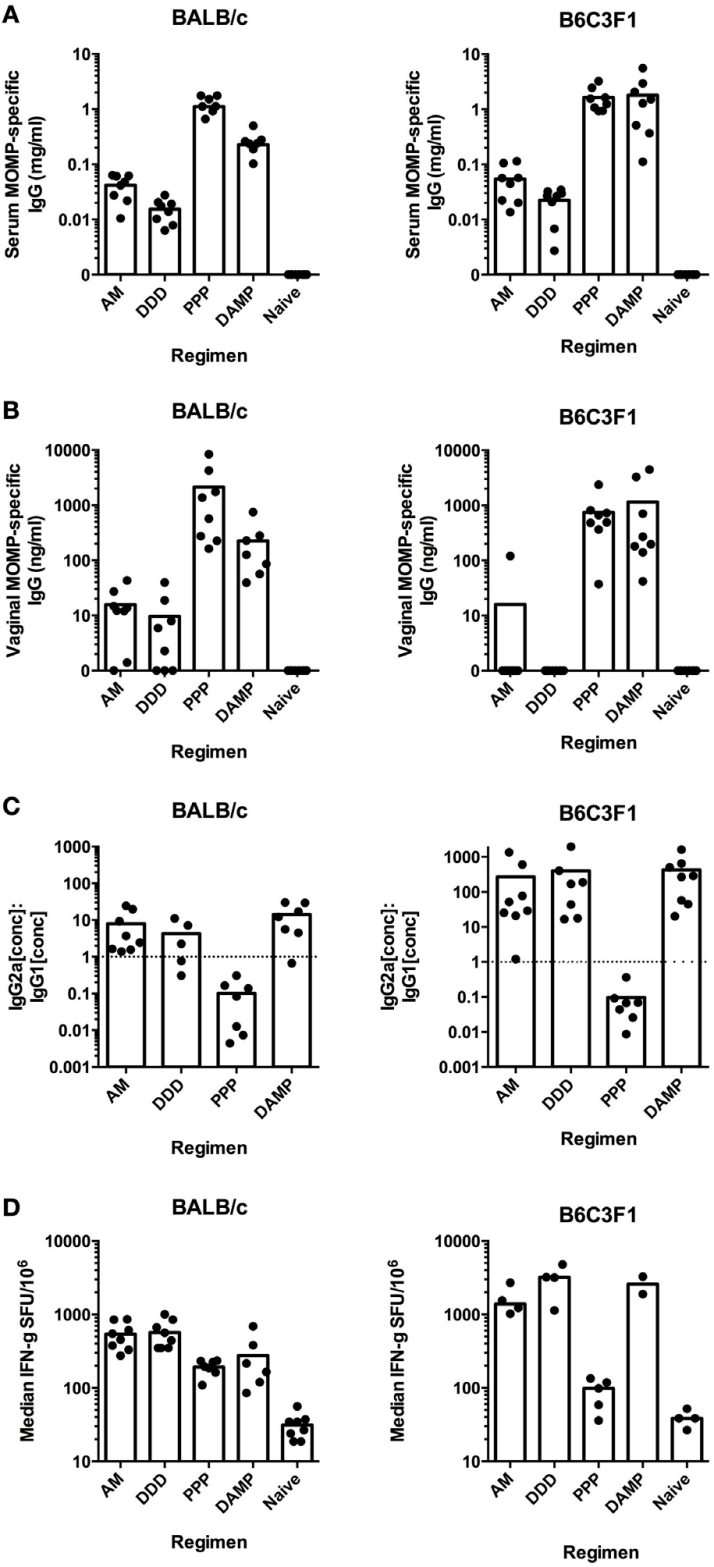
**Prime-boost vaccine regimens induce comparable immune responses in both BALB/c and B6C3F1 mouse strains**. MOMP-specific IgG concentrations were measured in serum **(A)** and vaginal washes **(B)** for both BALB/c and B6C3F1 vaccinated mice (*n* = 8 per group), expressed as individual concentrations with bars representing the means. **(C)** Serum MOMP-specific IgG2a and IgG1 isotype concentrations were measured by ELISA for both BALB/c and B6C3F1 vaccinated mice (*n* = 5–8 per group), and individual points and bars representing the means IgG2a:IgG1 ratios plotted. The dotted line indicates the IgG2a:IgG1 ratio of 1, demonstrating Th1-skewing above this line or Th2-skewing below it. **(D)** Splenocytes were assessed by IFN-γ ELISpot for MOMP-reactive T cells stimulated by a peptide pool consisting of 15-mers overlapping by 11 amino acids for both vaccinated BALB/c and B6C3F1 mice. Data expressed as individual values with bars representing group medians (+ interquartile range) (SFU/million antigen-stimulated cells).

As we wished to test the effect of altering the immune response on Chlamydial protection, the following groups were chosen for further investigation as they gave distinct, skewed immune responses in the BALB/c screen: AM and DDD (T cell, low antibody), PPP (Th2-skewed antibody, no Th1 T cells), and DAMP (Th1-skewed antibody and T cell). These vaccine regimens induced comparable responses in B6C3F1 mice as they did in BALB/c mice for MOMP-specific serum IgG concentrations (Figure 3A), MOMP-specific vaginal IgG concentrations (Figure 3B), MOMP-specific serum IgG2a to IgG1 ratios (Figure 3C), and MOMP-specific IFN-γ T-cell responses (Figure 3D).

### The DAMP Vaccine Regimen Enhances the Clearance of *C. trachomatis*, Regardless of Mouse Strain, and Is CD4^+^ T Dependent

**Figure 4:**
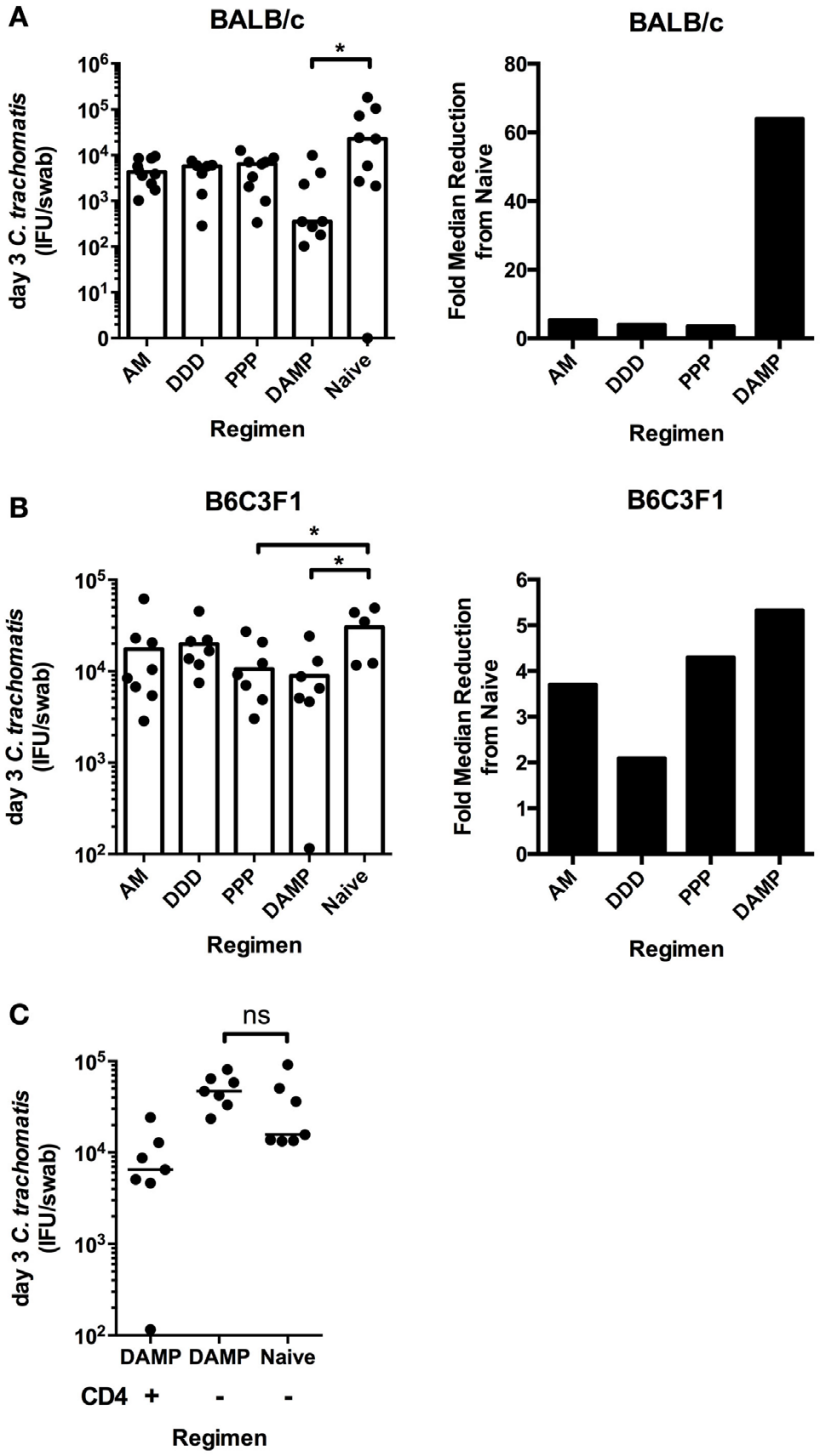
**The DAMP vaccine regimen enhances the clearance of intravaginal *C. trachomatis* in BALB/c and B6C3F1 mice, in a CD4^+^ T-cell-dependent manner**. Six weeks after the final vaccination and 1 week after 2 mg/mouse subcutaneous Depo-Provera treatment, BALB/c and B6C3F1 mice (*n* = 7–10 per group) were infected intravaginally with 4 × 10^5^ IFU of *C. trachomatis* D/UW-3/Cx. The vaginal vault of mice were sampled using individual swabs at day 3 [**(A)** BALB/c; **(B)** B6C3F1] after challenge, and vaginal Chlamydial loads quantified by infection assay and immunoflorescent microscopy. The fold reduction in median Chlamydial load compared to naive BALB/c **(A)** and B6C3F1 **(B)** mice at day 3 after infection is also represented. **(C)** B6C3F1 mice (*n* = 8 per group) were immunized with the DAMP regimen or left unvaccinated and subsequently depleted of CD4^+^ T cells by i.p. injections of 500 μg/mouse of anti-mouse CD4 monoclonal antibody (clone GK1.5) on days −1 and +1 with respect to day of challenge day 0. *C. trachomatis* load was measured in the vaginal vault at day 3 after infection. Individual and median values are represented. *p ≤ 0.05, **p ≤ 0.01, and ***p ≤ 0.001, two-tailed Mann–Whitney U test.

Immunized BALB/c mice were challenged with *C. trachomatis* D/UW-3/Cx intravaginally. The DAMP regimen significantly reduced chlamydial IFU per swab at day 3 after challenge (median = 354 IFU/swab) compared to unvaccinated controls (median = 22,688 IFU/swab) (DAMP: *p = 0.0359, two-tailed Mann–Whitney test) (Figure 4A). There were no statistical differences at the later sampling points of 7, 10, and 14 days (data not shown) after challenge reflecting the natural clearance of *C. trachomatis* in mice. The DDD, AM, and PPP regimens did not significantly reduce chlamydial shedding at any time points sampled after challenge in BALB/c mice.

Immunized B6C3F1 mice were challenged with *C. trachomatis* D/UW-3/Cx intravaginally. Similar to BALB/c mice, the DAMP vaccination regimen significantly reduced chlamydial IFU per swab at day 3 after challenge (median = 6,531 IFU/swab) compared to unvaccinated controls (median = 34,788 IFU/swab) (*p = 0.0303, two-tailed Mann–Whitney test) in B6C3F1s (Figure 4B). In addition, the PPP regimen significantly reduced chlamydial IFU per swab at day 3 after challenge (median = 8,095 IFU/swab) compared to unvaccinated controls (median = 34,788 IFU/swab) (*p = 0.0451, two-tailed Mann–Whitney test) (Figure 4B). There were no statistical differences at the later sampling points of day 7, day 10, or day 14 after challenge, and consistent with this challenge model, there was no oviduct pathology observed (hydrosalpinx) in challenged BALB/c or B6C3F1 mice (data not shown). Of the four tested prime-boost regimens, only the DAMP regimen consistently enhanced the clearance of intravaginal *C. trachomatis*.

We assessed the mechanism of the vaccine-induced enhanced clearance. Monoclonal antibodies were used to deplete CD4^+^ T cells in DAMP vaccinated and unvaccinated control B6C3F1 mice prior to intravaginal challenge. There was no difference between the DAMP immunized CD4^+^ depleted groups and the naive CD4^+^ depleted group in chlamydial loads, indicating that the DAMP vaccine-induced enhanced clearance is CD4^+^ T-cell dependent (Figure 4C).

## Discussion

New bioinformatic strategies have been developed as an approach to elicit broad immune responses against the more intractable antigenically variable pathogens, such as *C. trachomatis*. Two such approaches, consensus and mosaic, were used to design a MOMP antigen(s) to provide broad cellular cross-serovar coverage. We performed phylogenetic analyses revealing *C. trachomatis* MOMP to have quite distant species but high levels of amino acid conservation within a serovar. This phylogenetic structure lends itself more toward a consensus-based antigen design as opposed to a mosaic-based approach (8). Therefore, a single consensus MOMP antigen (Con E), constructed from variant sequences of the most common *C. trachomatis* serovar, was used. Nevertheless, combining the Con E antigen with additional mosaic antigens [that we have designed (Supplementary Material)] would provide complementary coverage to all other serovars. This would be a well-justified approach to theoretically achieve comprehensive coverage of all serovars in one vaccine, with no loss of coverage of the E serovar (Figure 1C), however assessing these additional antigens immunologically was out of the scope of this initial study.

Having designed our broad-coverage T-cell immunogen, we then assessed its immunogenicity using a range of prime-boost regimens. Few multi-component prime-boost vaccine regimens have been tested for the generation of immune responses against *Chlamydia* (46), with the majority focusing on homologous prime-boost strategies (47, 48). The use of different vectors within prime-boost regimen can help to avoid anti-vector immunity and improve vaccine-elicited immune responses. Previous uses of DNA vaccines expressing *Chlamydia* transgenes have not been adjuvanted by electroporation as in this study (49, 50), and only one previous use of an adenovirus-vectored vaccine against *Chlamydia muridarum* (expressing CPAF) has been reported (46).

Following an initial screen of 11 prime-boost regimens, 4 immunologically distinct regimens were assessed in both BALB/c and B6C3F1 mice. The AM, DDD, PPP, and DAMP regimens showed no mouse-strain-specific discrepancies in the humoral or cellular responses induced. Of these four regimens, only the DAMP vaccination regimen enhanced the clearance of intravaginal *C. trachomatis*, regardless of mouse strain. This enhanced chlamydial clearance afforded by the DAMP vaccination regimen was dependent on CD4^+^ T cells, as shown by CD4^+^ T-cell depletion following vaccination. Previous chlamydia vaccine studies in mice have also revealed a role for CD4^+^ T cells in their protection, including in the liposomal delivery of rMOMP (29) and the subcutaneous delivery of CTH1 (51).

The importance of IFN-γ *in vivo* to chlamydia control has been demonstrated previously (52, 53). In this respect, it appears somewhat surprising that the DDD and AM regimens, inducing stronger T cell IFN-γ responses than DAMP, failed to show any evidence for enhanced clearance. However, a distinct characteristic of the DAMP vaccination regimen was the co-induction of significantly higher levels of MOMP-specific IgG2a (with a high IgG2a/IgG1 ratio). This was in spite of the use of the protein-adjuvant MF59^®^, which has previously been shown to skew T-cell responses against MOMP toward IL-5 and IL-10 (44); this may be explained by the order of immunizations, with the initial MOMP antigen exposures in the context of DNA and viral vector delivery skewing the response, as the protein-adjuvant boosted regimens resulted in a strong IgG1 bias. Chlamydial-specific IgG2a antibody concentrations have previously correlated with protection in animal studies (3). Thus, although enhanced clearance was dependent upon CD4 T-cell responses, these data suggest that induced IgG2a responses may have played a contributory role in the enhanced clearance of *C. trachomatis* infection. In this respect, EB opsonization by IgG isotypes is known to affect the uptake of chlamydia into cells (54). EB opsonization with a high IgG1 and low IgG2a anti-MOMP polyclonal sera has been shown to enhance infection of a cell line through the FcRn-mediated uptake of these IgG-coated EBs, suggesting a negative role for MOMP-specific IgG1 antibodies (55). The IgG2a isotype by contrast mediates effector functions, including ADCC, with evidence suggesting this effector function may facilitate the early clearance of a chlamydial infection (15); furthermore, ADCC is associated with enhanced antigen presentation with the potential to amplify CD4 T-cell responses (56). Collectively, these suggest that the anti-MOMP IgG isotype may have a contributory role in the protection observed from the DAMP regimen, though without a DAM comparator arm this would require further investigation.

We cannot rule out that the DAMP vaccination regimen itself is in fact lowering the establishment of infection and, thus, resulting in the lower IFU observed, as our earliest sampling point is day 3 after challenge. Moreover, we cannot exclude the possibility that non-specific effects of the vaccine components in the DAMP regimen may impact general T-cell function. Future work should assess the potential non-specific immunologic effects of antigen-delivery by viral vectors alongside adjuvantation during prime-boost vaccinations.

In this study, we undertook a bioinformatic approach to generate an immunogen that would induce cross-serovar Chlamydial T-cell responses. We have revealed a capability to induce an array of MOMP-specific immune responses, both cellular and humoral, using four differing MOMP-based vaccine modalities in multi-component prime-boost regimens. The comparison of the same antigen by different modalities gives us insight into the distinct immune profiles induced by these vaccines. Based on our focus of developing vaccine candidates to progress toward clinical testing, we opted to use *C. trachomatis* for our challenged studies, rather than the more conventional use of *C. muridarum* that is pathogenic in mice. Though *C. trachomatis* is not a natural pathogen of mice, it has been argued that intravaginal infection with *C. trachomatis* mimics in many ways both the course and outcome of infection in most women as asymptomatic and self-limiting (57). In this context, the observed significant reduction in shedding following DAMP vaccination observed 3 days post infection is particularly encouraging given rapid natural clearance in naive animals. We would anticipate a greater impact on *C. trachomatis* clearance in transcervical infection models, in non-human primate models, and in humans, where the infection is slow to clear and/or may establish chronic infection and this will form the focus of our future studies.

## Author Contributions

AB-Z, PM, BK, GB, and AW performed the experiments; AB-Z, PM, JT, and RS designed the studies; AN, JG, and FF provided data sources. AB-Z and JT wrote the paper.

## Conflict of Interest Statement

The authors declare that the research was conducted in the absence of any commercial or financial relationships that could be construed as a potential conflict of interest.
