# Supplementary material for: A Multi-Component Prime-Boost Vaccination Regimen with a Consensus MOMP Antigen Enhances Chlamydia trachomatis Clearance
Source: Front Immunol. 2016 Apr 28;7:162. doi: 10.3389/fimmu.2016.00162 (PMC4848310; doi:10.3389/fimmu.2016.00162)
Supplement: Supplementary file 1 [file Data_Sheet_1.DOC]

**Supplementary File 1**

**Mosaic Antigen Design**

Within each serovar, MOMP sequences are highly conserved, with only sporadic substitutions. Because of the high degree of within-genotype conservation, when using a single consensus antigen the potential epitope coverage of the E serovar is extremely high (~95%) (Fig 1C-A), and so adding a second E mosaic to complement the first sequence is of marginal benefit (~98% coverage), certainly not enough to justify the additional cost of a second antigen. For bivalent and trivalent vaccine antigen designs across serovars, the mosaic vaccine designs can add value relative to consensus sequences in terms of providing better universal potential epitope coverage. Bivalent and trivalent mosaic designs extend the vaccine coverage to potential T cell epitopes from diverse lineages, and so we present these options as candidates for a second-generation antigen design. The D genotype, which is closest to genotype E, has only an average 68% of the potential epitopes matched, and all the other serovars have less than 50% potential epitope coverage if one only includes the con E antigen in a vaccine. Thus if one hopes to maximize the chances of a global vaccine that is effective against all serovars, ultimately a multivalent antigen may be necessary and so we also considered bivalent and trivalent options to best cover genital Chlamydia diversity (Fig 1C-B,C,D,E,F). There are several options for a bivalent approach to cover serovars D-K. One could mix two serovar consensus antigens, like the two common forms Con E and Con F. Coverage of serovars H-K would still be under 50% in this setting (Fig 1C-B). Alternatively, one could make a global 2 mosaic set (Fig 1C-C), which would bring up the overall level of coverage; in such a scenario, the most common E serovar would lose potential epitope coverage to accommodate improved overall coverage. Alternatively, one could fix the Con E, to maintain the excellent 95% E serovar coverage it provides, and resolve a mosaic complement, which results in no compromise in E and better global coverage of other serovars (Fig 1C-D). Finally, if the resources were available to make a trivalent vaccine, excellent coverage of all known serovars could be readily achieved. A trivalent mosaic solution is shown in Fig 1C-E, and a fixed serovar Con E with two complementary mosaics is shown in Fig 1C-F. The vaccine sequences that correspond to these various options are provided in the supplementary materials and can be used for second generation vaccine studies.

The results of the design options are nuanced, and the intent and target population of the vaccine balanced with production costs and resources would influence the selection of the most desirable constructs. Because within our experimental framework in this study we wanted to compare delivery strategies, we opted for a single Con E serovar sequence as our sole insert; this provides an excellent match to the most prevalent form of *C. trachomatis*. Having decided on a Con E MOMP antigen, this was inserted into DNA, HuAd5 and MVA based vaccine vectors, and the vaccines’ immunogenicity assessed in conjunction with an existing recombinant MOMP protein derived from *C. trachomatis* strain D/UW/Cx.

**Supplementary Methods and Materials:**

**Consensus antigen design**

The ConE antigen was created using the Advanced tool on Consensus Maker v2.0.1 (http://www.hiv.lanl.gov/content/sequence/CONSENSUS/consensus.htmL) using the following parameters: the minimum number of sequences for consensus was set to 3, with no consensus of consensuses; the unanimous value was set to 1.00, with a majority value of 0.50, using the most common character at each alignment point.

**Mosaic antigen design**

1,464 serovar E ompA sequences, surveyed from a total of 5026 C. trachomatis strains isolated in 33 distinct geographic regions from five continents were compared. 49 distinct ompA variants were present within the serovar E sequences, and were used in the in silico generation of both consensus and mosaic MOMP antigen sequences. The mosaic antigen generation included MOMP variants that included 20 sequences of each serovar to provide equal weight to each serovar for the mosaic sequence design. When only 10 distinct sequences from a serovar were available (serovars H and K) we used each one twice to increase their weight in the sample. We combined I with Ia, D with Da, and J with Ja as they were very similar, and then reduced all of the sets of greater than 20 sequences to 20 sequences by favouring inclusion of complete sequences, and randomly selecting from within the sets. Mosaic antigen sets were created using Mosaic Vaccine Designer, to either generate fully mosaic sets, or sets that included the Con E antigen as a fixed member of the set, and complementary mosaic antigens to best cover the other serovars (http://www.hiv.lanl.gov/content/sequence/MOSAIC/makeVaccine.htmL). The mosaic designs explored were based on a cocktail size set from between 1-3, with optimal epitope length of 9 (to allow the software to cover Class I and II), with the rare threshold value of 3. Default ‘Run settings’ included a Max Runtime of 5 hr, a population size of 200, with 10 cycles, stall factor of 10, internal crossover probability of 0.5 and iteration and random seed values of 0.

**EPICOVER analysis**

For the Epitope Coverage Assessment Tool EPICOVER (http://www.hiv.lanl.gov/content/sequence/MOSAIC/epicover.htmL) settings included a nominal epitope length of 9 for optimisation purposes (to allow the software to cover Class I and II), with a maximum amino acid mismatch of 2 in order to score, and a minimum of 3 occurrences of a potential epitope in the test set to consider for coverage. All analysis tools are available through web interfaces provided through the Los Alamos National Laboratory HIV database, www.hiv.lanl.gov.

**Antigen Sequences**:

>ConE
MKKLLKSVLVFAALSSASSLQALPVGNPAEPSLMIDGILWEGFGGDPCDP
CTTWCDAISMRMGYYGDFVFDRVLKTDVNKEFQMGDKPT-STTGNAT-AP
TTLTARENPAYGRHMQDAEMFTNAACMALNIWDRFDVFCTLGASSGYLKG
NSASFNLVGLFG-DNENQSTVKTNSVPNMSLDQSVVELYTDTAFSWSVGA
RAALWECGCATLGASFQYAQSKPKVEELNVLCNAAEFTINKPKGYVGQEF
PLALIAGTDAATGTKDASIDYHEWQASLALSYRLNMFTPYIGVKWSRASF
DADTIRIAQPKSATAIFDTTTLNPTIAGAGDV-KASAEGQLGDTMQIVSL
QLNKMKSRKSCGIAVGTTIVDADKYAVTVETRLIDERAAHVNAQFRF

>ConF
MKKLLKSVLVFAALSSASSLQALPVGNPAEPSLMIDGILWEGFGGDPCDP
CTTWCDAISMRMGYYGDFVFDRVLKTDVNKEFEMGEALA-GASGNTTSTL
SKLVERTNPAYGKHMQDAEMFTNAACMTLNIWDRFDVFCTLGATSGYLKG
NSASFNLVGLFG-DGVNATKPAADSIPNVQLNQSVVELYTDTTFAWSVGA
RAALWECGCATLGASFQYAQSKPKIEELNVLCNAAEFTINKPKGYVGKEF
PLDLTAGTDAATGTKDASIDYHEWQASLSLSYRLNMFTPYIGVKWSRASF
DSDTIRIAQPRLVTPVVDITTLNPTIAGCGSVAGANTEGQISDTMQIVSL
QLNKMKSRKSCGIAVGTTIVDADKYAVTVETRLIDERAAHVNAQFRF

>2mosaic.fixEcon.mos2
MKKLLKSVLVFAALSSASSLQALPVGNPAEPSLMIDGILWEGFGGDPCDP
CTTWCDAISMRMGYYGDFVFDRVLKTDVNKEFQMGAAPTTSDVEGLQNDP
TTNVARPNPAYGKHMQDAEMFTNAAYMALNIWDRFDVFCTLGATTGYLKG
NSASFNLVGLFGTKTQSSNFNTAKLIPNAALNQAVVELYTDTTFAWSVGA
RAALWECGCATLGASFQYAQSKPKIEELNVLCNAAEFTINKPKGYVGKEF
PLDLTAGTDAATGTKDASIDYHEWQASLALSYRLNMFTPYIGVKWSRVSF
DADTIRIAQPKLAEAILDVTTLNPTIAGKGTVVASGSDNDLADTMQIVSL
QLNKMKSRKSCGIAVGTAIVDADKYAVTVETRLIDERAAHVNAQFRF

>3mosaic.fixobothEconFcon.mos3
MKKLLKSVLVFAALSSASSLQALPVGNPAEPSLMIDGILWEGFGGDPCDP
CATWCDAISMRVGYYGDFVFDRVLKTDVNKEFQMGAAPTTSDVEGLQNDP
TTNVARPNPAYGKHMQDAEMFTNAAYMALNIWDRFDVFCTLGATTGYLKG
NSASFNLVGLFGTKTQSSNFNTAKLIPNAALNQAVVELYTDTAFAWSVGA
RAALWESGCPTLGASFQYAQSKPKVEELNVLCNASEFTINKPKGYVGAEF
PLDITAGTEAATGTKDASIDYHEWQASLSLSYRLNMFTPYIGVKWSRVSF
DADTIRIAQPKLAEAILDVTTLNPTIAGKGTVVASGSDNDLADTMQIVSL
QLTKMKSRKSCGIAVGTAIVDADKYAVTVETRLIDERAAHVNAQFRF

>3mosaic.fixEcon.mos2
MKKLLKSVLVFAALSSASSLQALPVGNPAEPSLMIDGILWEGFGGDPCDP
CTTWCDAISMRVGYYGDFVFDRVLKTDVNKEFEMGEALA-GASGNTTSTL
SKLVERTNPAYGKHMQDAEMFTNAACMTLNIWDRFDVFCTLGATSGYLRG
NSASFNLVGLFG-DGVNATKPAADSIPNVQLNQSVVELYTDTAFAWSVGA
RAALWESGCPTLGASFQYAQSKPKIEELNVLCNASEFTINKPKGYVGKEF
PLDLTAGTDAATGTKDASIDYHEWQASLALSYRLNMFTPYIGVKWSRASF
DSDTIRIAQPRLVTPVVDITTLNPTIAGCGSVAGANTEGQISDTMQIVSL
QLNKLKSRKSCGIAVGTAIVDADKYAVTVETRLIDERAAHVNAQFRF

>3mosaic.fixEcon.mos3
MKKLLKSVLVFAALSSASSLQALPVGNPAEPSLMIDGILWEGFGGDPCDP
CATWCDAISMRVGYYGDFVFDRVLKTDVNKEFQMGAAPTTSDVEGLQNDP
TTNVARPNPAYGKHMQDAEMFTNAAYMALNIWDRFDVFCTLGATTGYLKG
NSASFNLVGLFGTKTQSSNFNTAKLIPNAALNQAVVELYTDTTFAWSVGA
RAALWECGCATLGASFQYAQSKPKVEELNVLCNAAQFTINKPKGYVGAEF
PLDITAGTEAATGTKDASIDYHEWQASLSLSYRLNMFTPYIGVKWSRVSF
DADTIRIAQPKLAEAILDVTTLNPTIAGKGTVVASGSDNDLADTMQIVSL
QLTKMKSRKSCGIAVGTAIVDADKYAVTVETRLIDERAAHVNAQFRF

>2mosaic.seq1
MKKLLKSVLVFAALSSASSLQALPVGNPAEPSLMIDGILWEGFGGDPCDP
CATWCDAISMRVGYYGDFVFDRVLKTDVNKEFEMGEALA-GASGNTTSTL
SKLVERTNPAYGKHMQDAEMFTNAACMALNIWDRFDVFCTLGATSGYLRG
NSASFNLVGLFG-DGVNATKPAADSIPNVQLNQSVVELYTDTAFSWSVGA
RAALWESGCPTLGASFQYAQSKPKVEELNVLCNASEFTINKPKGYVGAEF
PLDITAGTEAATGTKDASIDYHEWQASLSLSYRLNMFTPYIGVKWSRASF
DSDTIRIAQPKSATAIFDTTTLNPTIAGCGSVAGANTEGQISDTMQIVSL
QLTKMKSRKSCGIAVGTTIVDADKYAVTVETRLIDERAAHVNAQFRF

>2mosaic.seq2
MKKLLKSVLVFAALSSASSLQALPVGNPAEPSLMIDGILWEGFGGDPCDP
CTTWCDAISMRMGYYGDFVFDRVLKTDVNKEFQMGAAPTTSDVEGLQNDP
TTNVARPNPAYGKHMQDAEMFTNAAYMALNIWDRFDVFCTLGATTGYLKG
NSASFNLVGLFGTKTQSSNFNTAKLIPNAALNQAVVELYTDTTFAWSVGA
RAALWECGCATLGASFQYAQSKPKIEELNVLCNAAEFTINKPKGYVGKEF
PLDLTAGTDAATGTKDASIDYHEWQASLALSYRLNMFTPYIGVKWSRVSF
DADTIRIAQPKLAEAILDVTTLNPTIAGKGTVVASGSDNDLADTMQIVSL
QLNKMKSRKSCGIAVGTAIVDADKYAVTVETRLIDERAAHVNAQFRF

>3mosaic.seq1
MKKLLKSVLVFAALSSASSLQALPVGNPAEPSLMIDGILWEGFGGDPCDP
CTTWCDAISMRVGYYGDFVFDRVLKTDVNKEFEMGEALA-GASGNTTSTL
SKLVERTNPAYGKHMQDAEMFTNAACMTLNIWDRFDVFCTLGATSGYLKG
NSASFNLVGLFG-DNENQKTVKAESVPNMSFDQSVVELYTDTAFSWSVGA
RAALWECGCATLGASFQYAQSKPKIEELNVLCNAAEFTINKPKGYVGKEF
PLDLTAGTDAATGTKDASIDYHEWQASLSLSYRLNMFTPYIGVKWSRASF
DSDTIRIAQPRLVTPVVDITTLNPTIAGCGSVAGANTEGQISDTMQIVSL
QLNKLKSRKSCGIAVGTTIVDADKYAVTVETRLIDERAAHVNAQFRF

>3mosaic.seq2
MKKLLKSVLVFAALSSASSLQALPVGNPAEPSLMIDGILWEGFGGDPCDP
CATWCDAISMRVGYYGDFVFDRVLETDVNKEFHMGAKPT--TDTGNSAAP
STLTARENPAYGRHMQDAEMFTNAACMALNIWDRFDVFCTLGATTGYLRG
NSASFNLVGLFG-DGVNATKPAADSIPNVQLNQSVVELYTDTAFAWSVGA
RAALWESGCPTLGASFQYAQSKPKVEELNVLCNAAQFTINKPKGYVGQEF
PLALIAGTDAATGTKDASIDYHEWQASLALSYRLNMFTPYIGVKWSRASF
DSNTIRIAQPKSATAIFDTTTLNPTIAGAGDV-KTGAEGQLGDTMQIVSL
QLTKMKSRKSCGIAVGTTIVDADKYAVTVETRLIDERAAHVNAQFRF

>3mosiac.seq3
MKKLLKSVLVFAALSSASSLQALPVGNPAEPSLMIDGILWEGFGGDPCDP
CTTWCDAISMRMGYYGDFVFDRVLKTDVNKEFQMGAAPTTSDVEGLQNDP
TTNVARPNPAYGKHMQDAEMFTNAAYMALNIWDRFDVFCTLGATTGYLKG
NSASFNLVGLFGTKTQSSNFNTAKLIPNAALNQAVVELYTDTTFAWSVGA
RAALWECGCATLGASFQYAQSKPKVEELNVLCNASEFTINKPKGYVGAEF
PLDITAGTEAATGTKDASIDYHEWQASLALSYRLNMFTPYIGVKWSRVSF
DADTIRIAQPKLAEAILDVTTLNPTIAGKGTVVASGSDNDLADTMQIVSL
QLNKMKSRKSCGIAVGTAIVDADKYAVTVETRLIDERAAHVNAQFRF
