# Supplementary figures and images for: A Multi-Component Prime-Boost Vaccination Regimen with a Consensus MOMP Antigen Enhances Chlamydia trachomatis Clearance
Source: Front Immunol. 2016 Apr 28;7:162. doi: 10.3389/fimmu.2016.00162 (PMC4848310; doi:10.3389/fimmu.2016.00162)

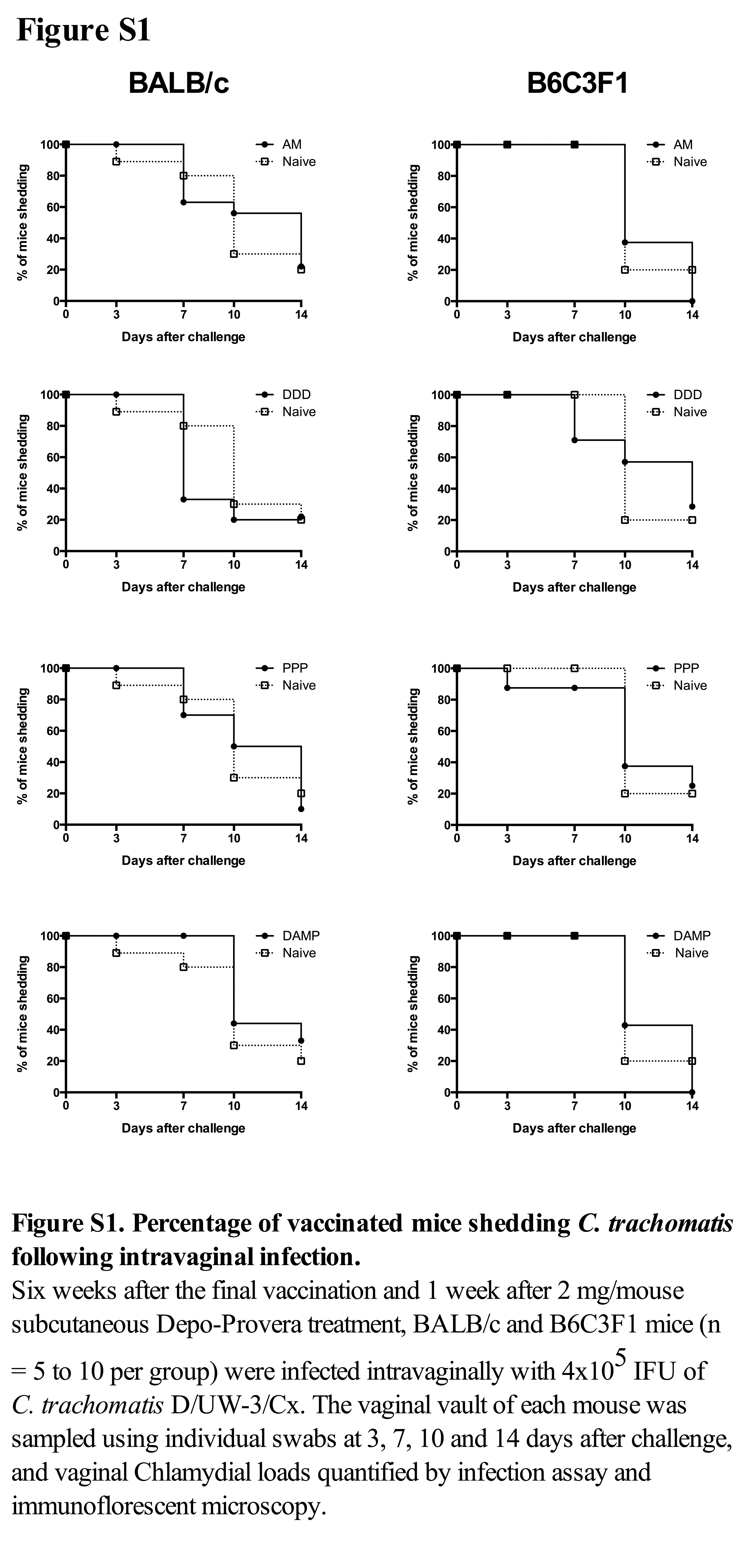

Supplement: Supplementary file 2 [file Image_1.JPEG]

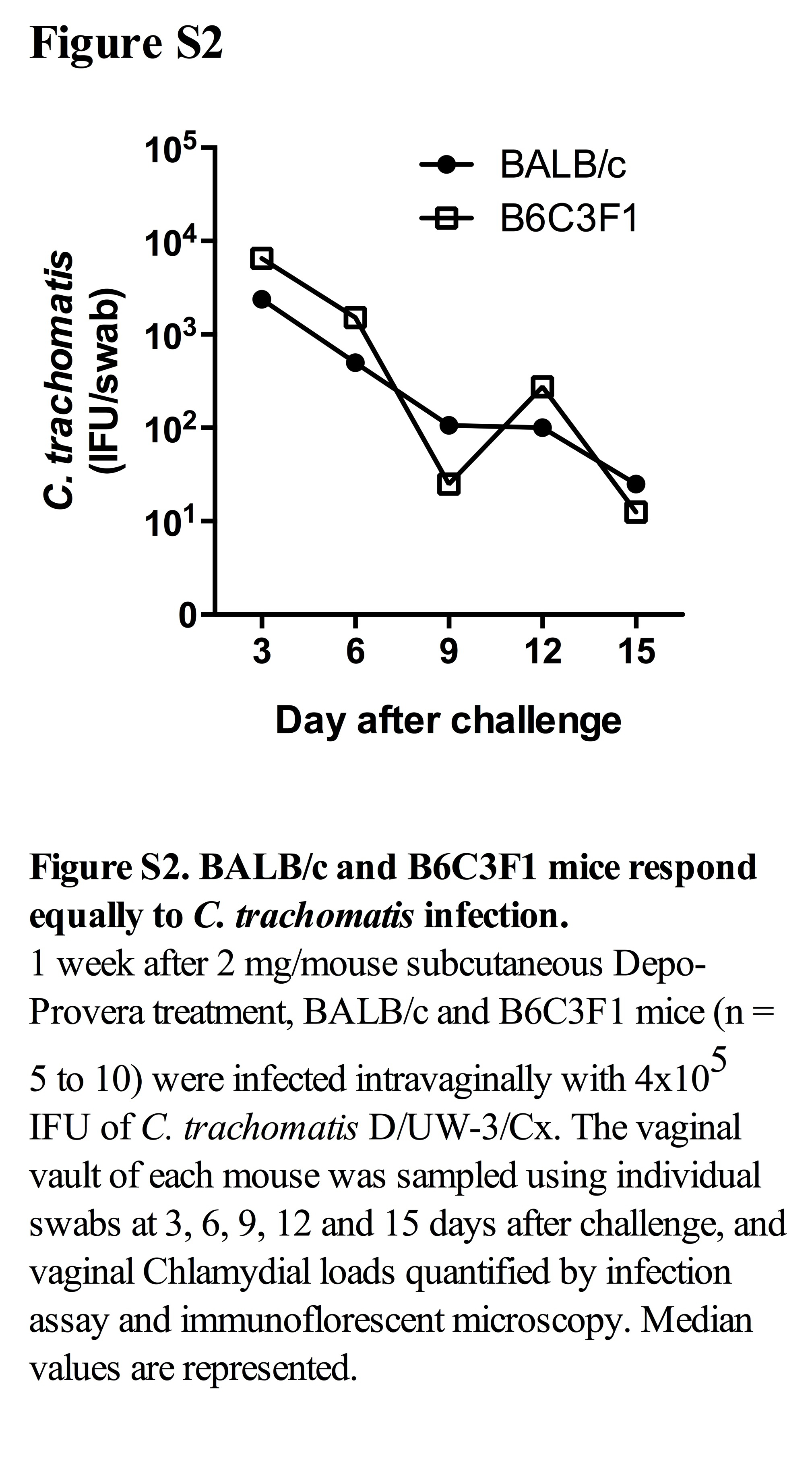

Supplement: Supplementary file 3 [file Image_2.JPEG]
